# Predictive Modeling of Poor Outcome in Severe COVID-19: A Single-Center Observational Study Based on Clinical, Cytokine and Laboratory Profiles

**DOI:** 10.3390/jcm10225431

**Published:** 2021-11-20

**Authors:** Óscar Gorgojo-Galindo, Marta Martín-Fernández, María Jesús Peñarrubia-Ponce, Francisco Javier Álvarez, Christian Ortega-Loubon, Hugo Gonzalo-Benito, Pedro Martínez-Paz, José Pablo Miramontes-González, Esther Gómez-Sánchez, Rodrigo Poves-Álvarez, Pablo Jorge-Monjas, Eduardo Tamayo, María Heredia-Rodríguez, Álvaro Tamayo-Velasco

**Affiliations:** 1 BioCritic, Group for Biomedical Research in Critical Care Medicine, 47005 Valladolid, Spain; ogorgojo@saludcastillayleon.es (Ó.G.-G.); mmartin.iecscyl@saludcastillayleon.es (M.M.-F.); alvarez@med.uva.es (F.J.Á.); christlord26@gmail.com (C.O.-L.); hgonzalob@saludcastillayleon.es (H.G.-B.); pedrojose.martinez@uva.es (P.M.-P.); rpoves@saludcastillayleon.es (R.P.-Á.); pjorgemo@saludcastillayleon.es (P.J.-M.); tamayo@med.uva.es (E.T.); maria_her_05@hotmail.com (M.H.-R.); alvarotv1993@gmail.com (Á.T.-V.); 2 Research Unit, University Clinical Hospital, 47003 Valladolid, Spain; 3 Department of Surgery, Faculty of Medicine, University of Valladolid, 47005 Valladolid, Spain; 4 Department of Medicine, Dermatology and Toxicology, Faculty of Medicine, University of Valladolid, 47005 Valladolid, Spain; 5 Centre for Biomedical Network Research on Infectious Diseases (CIBER), Carlos III Institute of Health, 28029 Madrid, Spain; 6 Haematology and Hemotherapy Department, University Clinical Hospital, 47003 Valladolid, Spain; mpenarrubia@saludcastillayleon.es; 7 Pharmacological Big Data Laboratory, Pharmacology Department, Faculty of Medicine, University of Valladolid, 47005 Valladolid, Spain; 8 Department of Cardiovascular Surgery, Clinic Hospital of Barcelona, 08036 Barcelona, Spain; 9 Institute of Health Sciences of Castile and Leon (IECSCYL), 47002 Soria, Spain; 10 IBSAL, Pontificia University of Salamanca, 37002 Salamanca, Spain; jpmiramontes@hotmail.com; 11 Internal Medicine Department, Rio Hortega University Hospital, 47012 Valladolid, Spain; 12 Anaesthesiology and Critical Care Department, University Clinical Hospital, 47003 Valladolid, Spain; 13 Anaesthesiology and Critical Care Department, University Clinical Hospital, 37007 Salamanca, Spain

**Keywords:** COVID-19, HGF, diagnosis, cytokines, biomarker, validation

## Abstract

Pneumonia is the main cause of hospital admission in COVID-19 patients. We aimed to perform an extensive characterization of clinical, laboratory, and cytokine profiles in order to identify poor outcomes in COVID-19 patients. Methods: A prospective and consecutive study involving 108 COVID-19 patients was conducted between March and April 2020 at Hospital Clínico Universitario de Valladolid (Spain). Plasma samples from each patient were collected after emergency room admission. Forty-five serum cytokines were measured in duplicate, and clinical data were analyzed using SPPS version 25.0. Results: A multivariate predictive model showed high hepatocyte growth factor (HGF) plasma levels as the only cytokine related to intubation or death risk at hospital admission (OR = 7.38, 95%CI—(1.28–42.4), *p* = 0.025). There were no comorbidities included in the model except for the ABO blood group, in which the O blood group was associated with a 14-fold lower risk of a poor outcome. Other clinical variables were also included in the predictive model. The predictive model was internally validated by the receiver operating characteristic (ROC) curve with an area under the curve (AUC) of 0.94, a sensitivity of 91.7% and a specificity of 95%. The use of a bootstrapping method confirmed these results. Conclusions: A simple, robust, and quick predictive model, based on the ABO blood group, four common laboratory values, and one specific cytokine (HGF), could be used in order to predict poor outcomes in COVID-19 patients.

## 1. Introduction

The COVID-19 pandemic is currently a major health and socioeconomic problem, with a crude mortality rate of about 2.3% [1,2]. The clinical spectrum of the disease ranges from subclinical infections to severe forms with pneumonia and shortness of breath, to multiorgan failure [3]. The facility of SARS-CoV-2 virus transmission (R(0) 2.2) between people results in an exponential increase in the number of hospitalization cases, with the consequent rapid occupation of intensive care units [4]. An early identification of poor prognosis factors is essential for the timely triaging of patients and resource optimization.

Several studies focused on the clinical characteristics and epidemiology of COVID-19 have suggested that specific risk factors increase the probability of infection with worse lung injury and death. Age, sex, smoking, hypertension, diabetes, and chronic cardiovascular and respiratory diseases are the risk factors best described in the literature and confirmed by many studies [5,6,7]. Several laboratory abnormalities have also been related to disease severity, such as high serum levels of C-reactive protein, procalcitonin, ferritin, and D-dimer levels [8]. Later, ABO blood groups were related to disease survival [9]. In fact, systematic reviews and meta-analyses have been carried out and concluded that blood groups A and B can be risk factors for a fatal outcome from COVID-19, whereas the blood group O appears to be protective [10,11,12]. In addition, cytokines, such as Hepatocyte Growth Factor (HGF), Interleukin (IL)-1α, and IL-27, appear to play an important role in severity or mortality risk [13]. In fact, recent publications have described the relationship between the O blood group and cytokine profile with lower rates of hospital admission and risk of intubation or death in COVID-19 patients [14].

Despite the fact that multiple characteristics have been associated with COVID-19 severity, the discovery of an effective therapeutic target remains unclear. However, the use of inhibitors of some cytokines (such as tocilizumab or anakinra) has not provided the expected results and there is disagreement on their effectiveness [15,16]. Thus, after a year of the current pandemic, perhaps the most important objective is to identify as soon as possible those patients with a higher risk of poor outcomes in order to provide early intensive treatments.

In this regard, the first purpose of this prospective and consecutive study was to carry out a broad characterization of the clinical characteristics, analytical values, and also levels of 45 cytokines in order to identify the factors involved in poor outcomes in COVID-19. Secondarily, we aimed to produce a reliable predictive model that can be used in emergency services to rapidly identify the patients who will need treatment with mechanical ventilation.

## 2. Materials and Methods

### 2.1. Study Subjects

This was a prospective and consecutive study of 108 patients with a positive PCR result for COVID-19 and admitted at Hospital Clínico Universitario de Valladolid (Spain) between 24 March and 11 April 2020. Patients with another active infection or any terminal chronic disease were excluded. Demographic, clinical, and analytical data were also obtained from each patient. This study was conducted according to the principles of Helsinki and approved by the Valladolid Hospital’s Clinical Ethics Committee (CEIm) (cod: PI 20-1717). It was also performed following the TRIPOD Statement.

### 2.2. Biological Samples

Plasma samples were obtained after emergency room admission in 3.2% sodium citrate tubes and centrifuged at 2000× *g* for 20 min at room temperature. The plasma was aliquoted and stored at −80 °C until used.

### 2.3. Blood Types

The blood group was determined using laboratory measurements using the samples in a fully automated analyzer (Erytra automated system for blood typing, Grifols, Barcelona, Spain) using DG gel card technology.

### 2.4. Variables

Demographic, clinical, and analytical data (leukocytes, lymphocytes, neutrophils, platelets, bilirubin, creatinine, glucose, high-sensitivity troponin-T (hs-TnT), C-reactive protein (CRP), lactate dehydrogenase (LDH), ferritin, procalcitonin, (PCT) and D-dimer of each patient were also recorded to describe the clinical phenotype.

### 2.5. Cytokine and Chemokine Analysis

Serum cytokines were tested using the 45-plex Human XL Cytokine Luminex Performance Panel (R&D). Cytokines and chemokines included in the panel were BDNF, EGF, eotaxin (also known as CCL11), FGF-2, GM-CSF, GRO-α (CXCL1), HGF, IFN-α, IFN-γ, IL-1α, IL-1β, IL-10, IL-12 p70, IL-13, IL-15, IL-17a (CTLA-18), IL-18, IL-1RA, IL-2, IL-21, IL-22, IL-23, IL-27, IL-31, IL-4, IL-5, IL-6, IL-7, IL-8 (also known as CXCL8), IL-9, IP-1 beta (CCL4), IP-10 (CXCL10), LIF, MCP-1 (CCL2), MIP-1α (CCL3), NGF-β, PDGFBB, PIGF-1, RANTES (CCL5), SCF, SDF-1α, TNF-α, TNF-β, VEGF-A, and VEGF-D. All cytokines, except for those underlined, had at least 20% detection to ensure the robustness of the results and were expressed in logarithm base 2.

### 2.6. Hospital Protocol Treatment

The hospital protocol for the treatment of COVID-19 pneumonia in March and April 2020 included: lopinavir/ritonavir (Kaletra^®^, Abbott, Chicago, IL, USA), 200/50 mg/mL solution taken twice a day, and hydroxychloroquine (Dolquine^®^, Rubió, Barcelona, Spain), 400 mg taken twice a day. According to inflammatory criteria, treatment could also include interferon 1β (Betaferon^®^, Bayer, Leverkusen, Germany), 0.25 mg taken every 48 h, corticosteroids (Urbason^®^, Aventis, Paris, France), 240 mg taken every day for three days, tocilizumab (RoActemra^®^, Roche, Basel, Switzerland), baricitinib (Olumiant^®^, Lilly, Indianapolis, IA, USA) or anakinra (Kineret^®^, Amgen, Thousand Oaks, CA, USA). In case of suspected bacterial superinfection, antibiotic treatment is required. Oxygen support (nasal cannula, high flow nasal cannula, and non-invasive or invasive mechanical ventilation) was administered to patients based on the severity of hypoxemia.

### 2.7. Statistical Analysis

In this study, which analyzed the relationship between clinical and analytical characteristics and cytokine profile with severity, the patients were divided into two study groups: intubated or dead and those who did not have this condition.

Data are presented as the median and interquartile range (IQR) for continuous data. The statistics for categorical variables are counts and percentages. The Mann–Whitney U test was performed for continuous variables, and the χ^2^ test and Fisher exact test were used for categorical variables, as appropriate.

Univariable binary logistic regression analyses, adjusted by age and gender, were used to preliminarily assess the association between the clinical, analytical, and cytokine variables with the dependent variable of severity. Subsequently, we obtained the cut-off values of the significant variables from the univariate analysis (*p* < 0.1) of the Receiver Operating Characteristic (ROC) curve with optimal sensitivity and specificity. Then, the mentioned variables were dichotomized and introduced in the multivariate analysis. Likewise, the resulting multivariate model was validated through the Leave One Out Cross-Validation (LOOCV) method with a resampling of 1000 individuals and an ROC curve based on model probabilities. The odds ratio (OR) and 95% Confidence Interval (CI) were reported.

All statistical analyses were performed using SPSS version 25.0 for Windows (SPSS, Inc., Chicago, IL, USA). A *p*-value of less than 0.05 was regarded as statistically significant.

## 3. Results

### 3.1. Presenting Characteristics

In total, 108 patients were hospitalized at Hospital Clínico Universitario de Valladolid with confirmed COVID-19 between 24 March and 11 April 2020. Clinical characteristics depending on the outcome (intubation or death) are shown in Table 1. There were no differences in age or sex. In comparison with other ABO blood types, an O blood type was associated with a significantly lower risk of intubation or death (*p* = 0.035). The most frequent comorbidity in both groups was hypertension; however, obesity was the only one that showed a significant difference (*p* = 0.023), being approximately four times more common in the intubated/deceased group. Among the laboratory findings, glycemia, creatinine, leukocytes, neutrophils, procalcitonin, ferritin, D-dimer, and LDH were significantly higher in the intubated/deceased group, while the total lymphocyte count was lower in this group. Regarding hospital meters, we observed that patients with worse outcomes had a longer hospital stay. Intubated patients in Intensive care unit (ICU), of which there were 32, had a 37.5% chance of 28-day mortality (12 patients). We also found 20 total deaths in the whole sample, which means that 60% (12/20) of deaths occurred in ICUs.

### 3.2. Cytokine Profile Comparison

**Figure 1:**
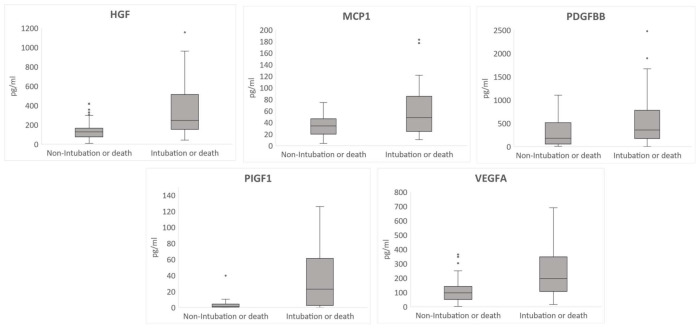
Box plot diagram showing the statistically significant cytokine levels according to intubation or death. Hepatocyte Growth Factor (HGF), Monocyte Chemoattractant Protein-1 (MCP1), Human Platelet-Derived Growth Factor-BB (PDGFBB), Human Placental Growth Factor 1 (PIGF1), Vascular Endothelial Growth Factor A (VEGFA). The dots on the box plot diagrams indicate the outlier values.

From the 37 cytokines that had the 20% minimum detection rate required, there were only six cytokines that showed statistically significant differences between groups (Table S1). IL-15 was the only cytokine for which plasma levels were lower in the group with the worst clinical outcomes. Conversely, HGF, MCP1, PDGFBB, PIGF1, and VEGFA were over-expressed in the intubated/deceased group (Figure 1). Levels were approximately double in the case of the cytokines HGF, PDGFBB, and VEGFA and six times more in the case of the cytokine PIGF1.

### 3.3. Risk Factors Associated with Poor Outcomes (Intubation or Death) in Hospitalized Patients with COVID-19

A univariate analysis of each clinical, analytical, and cytokine variable was performed (Tables S2 and S3). Among the significant clinical variables, three showed statistically significant results. The O blood group had a protective association (OR = 0.746), while diabetes (OR = 2.97) and obesity (OR = 5.36) were directly related to high intubation or mortality risk from COVID-19. Moreover, glycemia, leukocytes, neutrophils, procalcitonin, CRP, ferritin, D-dimer, and LDH were the analytical variables that were significantly associated with the worst outcomes, i.e., with the risk of intubation or death. Regarding the cytokine profile, four of the six cytokines that were significant in the previous comparison were associated with the study variable. HGF was the cytokine that showed the highest risk in the univariate analysis (OR = 2.12), followed by MCP1 (OR = 1.6) and PDGFBB (OR = 1.21). IL-15 was the only cytokine with an OR lower than 1, i.e., levels in the study group were lower than in patients who did not have poor clinical outcomes.

**Figure 2:**
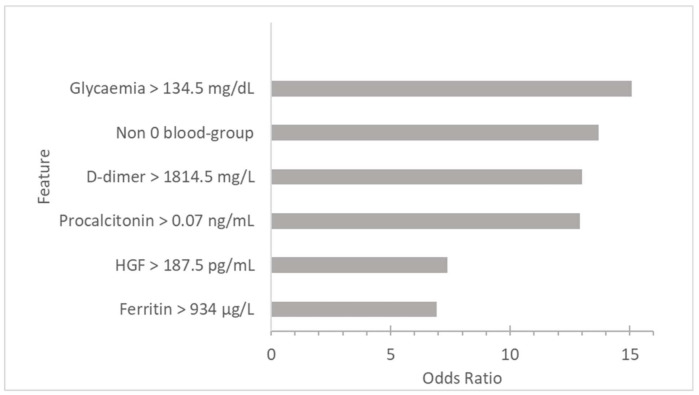
Multivariate analysis showing the contribution of each risk factor to intubation or death risk.

In the logistic regression, the significant continuous variables usually showed an OR of around 1 due to differences in the qualitative variables such as obesity and diabetes. In order to provide applicable results for emergency services, the significant continuous variables were dichotomized (Table 2). From the dichotomized variables, a multivariate model was made following an automatic backward step method, shown in Table S4 and represented in Figure 2. The O blood group was the only significant non-modifiable variable in the multivariate model (OR = 0.073) and converted the non-O blood groups to the second most important risk factor (OR = 13.7). The abnormal values of glycemia (OR = 15.09), D-dimer (OR = 13), procalcitonin (OR = 12.92), and ferritin (OR = 6.91) were the analytical variables that, above the cut-off value at the beginning of the infection, showed a higher risk of poor outcome. The only cytokine that had was statistically significant was HGF (OR = 7.38), with similar repercussions to the analytical variables.

**Figure 3:**
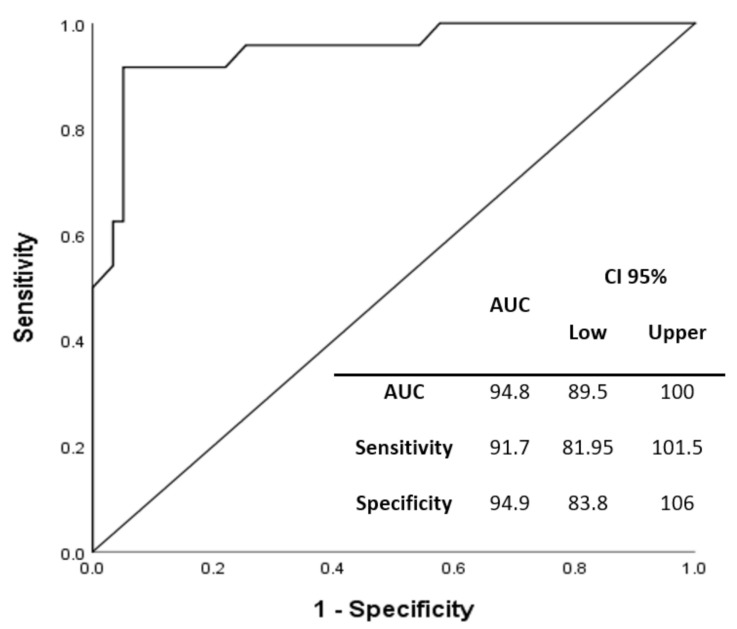
Internal validation using the AUC (area under the ROC curve). CI—confidence interval.

Internal validation was performed using the AUC to determine the sensitivity and specificity of the multivariate model (Figure 3). The model showed an accuracy of 94.8%, a sensitivity of 91.7%, and a specificity of 95%. Moreover, we also performed another internal validation following the bootstrapping method (Table S5). This retro-validation took into account a sample of 1000 patients and showed statistically significant results in the six variables included in the multivariate predictive model (O blood-group *p* = 0.031; glycaemia *p* = 0.003; procalcitonin *p* = 0.001; ferritin *p* = 0.013; D-dimer *p* = 0.008; HGF *p* = 0.003) and had low bias by addressing optimism and overfitting. It confirmed the solid and robust previous results.

## 4. Discussion

In this study, we performed an extensive characterization of clinical, laboratory, and cytokine profiles in order to identify poor outcomes from COVID-19. In this regard, after an analysis of 45 molecules, HGF was the unique cytokine able to predict a poor prognosis, defined as intubation or death (OR = 7.38; 95%CI (1.28–42.4); *p* = 0.025). The ABO blood group was an important characteristic; in fact, O blood group patients showed a lower risk of poor outcome (OR = 0.073; 95%CI (0.008–0.654); *p* = 0.019) when there were no comorbidities clearly involved. Finally, a multivariate predictive model including simple variables such as procalcitonin, ferritin, and D-dimer, along with ABO blood group and HGF, was performed and showed an accuracy of 94%, validated internally and by a bootstrapping method.

Cytokines play an important role in the immunopathology of viral infections, including COVID-19. Moderate cytokine production during SARS-CoV-2 infection gives rise to mild flu-like symptoms (e.g., fever, cough, difficulty breathing, fatigue, muscle or body aches, and headache etc.) and the body launches compensatory or repair processes to restore tissue and organ function [17]. However, it is well-known that a SARS-CoV-2 infection can also trigger an uncontrolled inflammatory response that gives way in the most severe cases to a cytokine storm and the resulting pathological inflammatory response leads to collateral damage in tissues and therefore a fatal clinical outcome [18,19]. Much of the damage caused to tissues is due to an imbalance in the recruitment of immune cells that leads to a decrease in lymphocytes (lymphopenia) and an increase in activated neutrophils [20,21]. It is for this reason that interesting studies have been published that show how the neutrophil-to-lymphocyte ratio (NLR), combined with IgG, could be used to indicate the severity of COVID-19 [22,23]. Recent studies have focused on the role of neutrophils in the context of hyper inflammation during infection by SARS-CoV-2 with the most critical cases; it has been observed that the cytokine HGF, contained in the granules of neutrophils, has an important predictive value in the severity of the disease [24,25,26]. Our study supports the notion that the levels of HGF may have a predictive value in the risk of intubation or death. Possibly, the release of the cytokine granulocyte colony-stimulating factor (G-CSF) stimulates an increase in the count of neutrophils in the blood, which are recruited to the lungs, where other cytokines activate them to release the contents of their granules [27].

To date, one of the best-studied aspects of COVID-19 has been the potential risk factors that affect susceptibility to infection and disease progression. The clinical (e.g., hypertension, diabetes, and chronic cardiovascular and respiratory diseases) and sociodemographic (e.g., sex, age, and race/ethnicity) aspects of predicting severe outcomes have already been established [28]. During the past 2020, the ABO blood group has been the subject of intense research due to its involvement in susceptibility to many pathogens, including SARS-CoV [29,30]. Growing evidence suggests that the ABO blood group may also play a role in the immunopathogenesis of SARS-CoV-2 infection, with group O being protective and group A conferring risk of greater disease susceptibility and severity [31]. Possibly, the differences in blood group antigen expression can increase or decrease the sensitivity of the host to pathogen infection, which may partially explain the blood group difference affecting host predisposition to COVID-19 [32]. However, another possible explanation contemplates the ability of anti-A antibodies to inhibit the interaction between the S protein and the ACE2 receptor, providing protection [30].

The identification of laboratory biomarkers to predict the severity of COVID-19 would allow for the early identification of unfavorable cases and effectively provide mechanical ventilation treatment. It is well known that the severity of the disease is associated with elevated levels of C-reactive protein (CRP), procalcitonin (PCT), D-dimer, or ferritin [8]. Recently, predictive and validated models based on univariate logistic regressions have been proposed in clinical practice, providing a criterion to diagnose the COVID-19-associated cytokine storm at an early stage [33,34]. Our study broadens the perspective of a predictive model in the diagnosis of poor outcomes in COVID-19 disease through multivariate analysis and taking into account not only laboratory parameters, but also the cytokine profile of each patient in the study. Furthermore, our study agrees with the data available and we provide cut-off values that contribute to obtaining the high sensitivity and specificity of our model.

The main limitations of our study are related to the relatively small number of patients included in the study with a small representation of common diseases in aging populations, the fact that it was a single-center study, and finally, the fact that some measurements, such as the concentration of procalcitonin and HGF, exceed the capabilities of the average hospital that is currently struggling with COVID-19. However, data collection and quantification of cytokines has been very extensive and rigorous, which supports the reliability of the study. In addition, it is necessary to further study the origin of HGF because a direct relationship with neutrophils has not been proven. There is also the possibility that HGF is indirectly related to the pathophysiology of liver dysfunction and the cytokine storm in the severe cases of COVID-19.

## 5. Conclusions

To conclude, our results show that several known risk factors, together with one of the thirty-seven cytokines analyzed (HGF), constitute a model capable of predicting with excellent sensitivity and specificity those patients with a worse prognosis. The determination of cytokine levels is not routinely performed in emergency services, but the rest of the analytical parameters are currently assessed in COVID-19 patients. It is interesting that the determination of a single cytokine and knowing the blood group could greatly help to improve how these patients should be treated, since the cut-off values of glycemia, procalcitonin, ferritin, and D-dimer are frequently exceeded in COVID-19 patients.

## Figures and Tables

**Table 1:** Clinical characteristics of the patients according to Intubation or death patients.

	Intubation or Death (*n* = 40)	Non-Intubation or Death (*n* = 68)	*p*
Age. Years (median (IQR))	72.5 (15.25)	72.5 (16.75)	0.195
Male (*n* (%))	22 (55)	37 (54.4)	0.953
**-Blood group (*n* (%))**			
O Blood group	8 (20)	27 (39.7)	0.035
**-Comorbidities (*n* (%))**			
Smoking	4 (10)	5 (7.4)	0.631
Coronary disease	4 (10)	6 (8.8)	0.839
Atrial fibrillation	5 (12.5)	7 (10.3)	0.725
Diabetes	1 (2.5)	0 (0)	0.190
Neurological disease	1 (2.5)	1 (1.5)	0.702
Stroke	0 (0)	1 (1.5)	0.441
Hypertension	20 (50)	30 (44.1)	0.554
Liver disease	2 (5)	0 (0)	0.063
Obesity	7 (17.5)	3 (4.4)	0.023
COPD	3 (7.5)	4 (5.9)	0.742
Kidney disease	2 (5)	1 (1.5)	0.281
**-Laboratory (median (IQR))**			
Glycaemia (mg/dL)	174.5 (103.75)	96 (35)	<0.001
Creatine (mg/dL)	0.9 (0.56)	0.81 (0.21)	0.042
Total bilirubin (mg/dL)	0.5 (0.57)	0.5 (0.3)	0.292
Leukocytes (×10^9^/L)	7.87 (7.83)	6.12 (3.45)	0.001
Lymphocytes (×10^9^/L)	605 (552.5)	1000 (512.5)	<0.001
Neutrophil (×10^9^/L)	6.74 (7.38)	4.19 (2.99)	<0.001
Procalcitonin (ng/mL)	0.23 (0.4)	0.06 (0.14)	<0.001
Platelet (×10^9^/L)	211.5 (107.5)	199 (115)	0.611
CRP (mg/L)	97 (153)	78 (99.75)	0.166
Ferritin (µg/L)	1456 (1246.5)	646 (864.75)	0.003
D-dimer (mg/L)	1594.5 (9282)	630 (564.5)	<0.001
LDH (mmol/L)	365 (179)	300 (720)	<0.001
**-Hospital meters (median (IQR))**			
Length of hospital stay (days)	22 (28)	8 (6)	<0.001
Length of ICU stay (days)	14 (13)	0 (0)	

Continuous variables are represented as [median (interquartile range—IQR)]; categorical variables are represented as [*n* (%)]. COPD—chronic obstructive pulmonary disease; CRP—C-reactive protein; ICU—intensive care unit; LDH—lactate dehydrogenase.

**Table 2:** Estimation of the cut-off value of the significant analytical variables by ROC curve.

	Cut-Off Value	Reference Value	Sensitivity (%)	Specificity (%)	AUC	CI 95%	
						Low	High
Glycaemia	134.5 mg/dL	70–110	82.5	85.3	89	82.5	95.5
Creatine	1.19 mg/dL	0.7–1.1	32.5	91.2	61.7	50.5	72.9
Leukocytes	9.94 × 10^9^/L	4.5–11.5	37.5	95.6	68.4	57.5	79.4
Lymphocytes	0.8 × 10^9^/L	1.3–4	0.05	98.5	23.2	0.128	0.336
Neutrophil	5.48 × 10^9^/L	2–7.5	67.5	73.5	75.2	65.3	85.1
Procalcitonin	0.07 ng/mL	<0.1	97.4	54.8	78.2	69.3	87.1
CRP	145 mg/L	<10	38.5	80.9	58.1	46.5	69.6
Ferritin	934 µg/L	<307	72	61.8	70.3	57.4	83.2
D-dimer	1814.5 mg/L	<120	67	78.5	74.6	64	85.3
LDH	326 nmol/L	<225	70	76.5	71.5	61.1	82
HGF	187.5 pg/mL	-	72.5	72.1	75.2	65.7	84.8
IL-15	29.6 pg/mL	-	22.5	80.9	39	27.4	50.7
MCP1	56.77 pg/mL	-	42.5	86.8	62.6	51.2	74
PDGFBB	182.5 pg/ml	-	77.5	52.9	61.7	50.7	72.8

AUC—area under the Receiver Operating Characteristic (ROC ) curve; CI—confidence interval.

## Data Availability

The datasets generated during and/or analyzed during the current study are available from the corresponding author on reasonable request.

## Supplementary Materials

The following are available online at https://www.mdpi.com/article/10.3390/jcm10225431/s1, Table S1: Comparison of the cytokine profile. Table S2: Individual logistic regression model for each cytokine adjusted by gender and age. Table S3: Individual logistic regression model for each clinical characteristic adjusted by gender and age. Table S4: Backward logistic regression. Table S5: Bootstrap for Variables in the Equation.
