# Supplementary material for: Predictive Modeling of Poor Outcome in Severe COVID-19: A Single-Center Observational Study Based on Clinical, Cytokine and Laboratory Profiles
Source: J Clin Med. 2021 Nov 20;10(22):5431. doi: 10.3390/jcm10225431 (PMC8622763; doi:10.3390/jcm10225431)
Supplement: Supplementary file 1 [file jcm-10-05431-s001.zip › jcm-1458578-supplementary.pdf]

Supplementary Materials:

Table S1. Comparison of the cytokine profile.

	**Intubation or death (N = 40)**	**Non-Intubation or death (N = 68)**	***P* value**
BDNF	59.88 (108.75)	60.38 (184.53)	0.967
EGF	2.36 (4.37)	1.88 (8.49)	1.000
Eotaxin	13.58 (8.54)	15.03 (10.22)	0.410
FGF2	1.07 (1.92)	0.80 (2.91)	0.879
GMCSF	11.26 (15.29)	13.09 (34.34)	0.397
GROa	2.82 (3.83)	3.48 (4.31)	0.346
HGF	294.75 (486.25)	139.25 (142.43)	<0.001
IFNa	0.45 (1.33)	0.58 (2.14)	0.712
IFNg	9.72 (11.17)	8.29 (6.84)	0.383
IL1a	3.37 (10.83)	1.81 (7.58)	0.127
IL1b	6.31 (10.72)	6.86 (10.09)	0.208
IL10	1.8 (2.85)	1.73 (2.02)	0.911
IL12p70	3.73 (2.62)	3.32 (3.25)	0.985
IL13	2.19 (2.55)	2.06 (3.46)	0.672

IL15	9.55 (19.65)	13.83 (17.47)	0.058
IL17a	6.35 (18.35)	7.32 (12.06)	0.303
IL18	58.98 (60.39)	40.28 (48.09)	0.160
IL1RA	644.67 (1312.68)	604.75 (961.67)	0.904
IL2	10.91 (17.42)	14.64 (20.59)	0.185
IL21	2.85 (7.97)	3.70 (13.81)	0.370
IL22	6.62 (46.36)	2.79 (20.08)	0.257
IL23	7.99 (7.58)	7.67 (10.50)	0.717
IL27	19.43 (26.49)	18.06 (44.09)	0.755
IL31	5.65 (6.44)	5.01 (8.85)	0.741
IL4	5.60 (4.95)	5.76 (7.88)	0.851
IL5	5.12 (36.16)	6.1 (16.84)	0.884
IL6	9.06 (28.74)	15 (20.69)	0.270
IL7	1.57 (3.01)	1.69 (3.01)	0.977
IL8	2.08 (4.61)	2.06 (3.57)	0.705
IL9	2.21 (2.81)	2.03 (4.35)	0.834
IP1b	53.33 (40.05)	45 (47.97)	0.794
IP10	48.78 (46.14)	43 (36.14)	0.390
LIF	13.48 (20.55)	15.70 (16.11)	0.997
MCP1	48.85 (62.81)	35.97 (28.01)	0.029
MIP1a	3.31 (12.61)	3.39 (10.98)	0.760

NGFb	4.36 (2.91)	4.08 (3.59)	0.975
PDGFBB	409.08 (1037.63)	203 (591.37)	0.042
PIGF1	27.22 (116.59)	4.24 (48.78)	0.036
RANTES	23.43 (17.75)	21.54 (19.79)	0.942
SCF	8.42 (9.50)	6.51 (5.98)	0.408
SDF1a	713.92 (742.29)	643 (500.75)	0.441
TNFa	7.56 (11.04)	5.85 (11.04)	0.736
TNFb	3.31 (3.58)	3.08 (5.33)	0.804
VEGFA	205.5 (250.56)	105.66 (162.2)	0.012
VEGFD	11.3 (13.23)	12.90 (13.34)	0.185

Continuous variables are represented as [median (interquartile range—IQR)].

Table S2. Individual logistic regression model for each cytokines adjusted by gender and age.

**cytokine**	***p*-value**	**OR**	**CI 95%**	
			**Low**	**High**
BDNF	0.910	0.988	0.808	1.210
EGF	0.841	0.984	0.838	1.155
Eotaxin	0.666	0.888	0.516	1.526
FGF2	0.882	0.987	0.828	1.176
GMCSF	0.336	0.894	0.711	1.124
GROa	0.410	0.888	0.669	1.178
HGF	0.000	2.121	1.468	3.062
IFNa	0.933	1.006	0.870	1.165
IFNg	0.303	1.230	0.830	1.822
IL1a	0.166	1.081	0.968	1.208
IL1b	0.117	0.789	0.586	1.061
IL10	0.947	1.013	0.696	1.474
IL12p70	0.887	1.035	0.639	1.677
IL13	0.608	0.941	0.746	1.187
IL15	0.026	0.696	0.506	0.958
IL17a	0.267	0.872	0.685	1.110

IL18	0.222	1.212	0.890	1.652
IL1RA	0.993	0.999	0.830	1.203
IL2	0.135	0.768	0.544	1.085
IL21	0.391	0.947	0.838	1.072
IL22	0.401	1.038	0.951	1.133
IL23	0.722	0.943	0.683	1.302
IL27	0.704	0.970	0.831	1.133
IL31	0.754	0.956	0.723	1.265
IL4	0.907	0.982	0.730	1.322
IL5	0.693	1.029	0.894	1.184
IL6	0.241	0.858	0.664	1.108
IL7	0.988	0.998	0.769	1.296
IL8	0.567	0.949	0.792	1.136
IL9	0.974	1.005	0.752	1.342
IP1b	0.733	0.946	0.688	1.301
IP10	0.713	1.064	0.766	1.477
LIF	0.909	1.019	0.739	1.405
MCP1	0.022	1.598	1.071	2.384
MIP1a	0.994	0.999	0.830	1.203
NGFb	0.582	1.150	0.699	1.890
PDGFBB	0.053	1.210	0.998	1.467

PIGF1	0.124	1.074	0.981	1.177
RANTES	0.478	1.154	0.777	1.713
SCF	0.452	1.148	0.801	1.645
SDF1a	0.686	1.040	0.861	1.256
TNFa	0.974	0.996	0.759	1.305
TNFb	0.979	1.004	0.716	1.409
VEGFA	0.123	1.180	0.956	1.456
VEGFD	0.113	0.739	0.508	1.075

OR—Odss Ratio; CI—Confidence interval.

Table S3. Individual logistic regression model for each clinical characteristic adjusted by gender and age.

Clinical characteristics	*P* value	OR	CI 95%	
			Low	High
**Blood Group**				
O Blood Group	0.061	0.746	0.549	1.013
**Comorbidities**				
Smoking	0.622	1.428	0.347	5.880
Coronary disease	1.000	1.000	0.255	3.919
Atrial fibrillation	0.916	0.929	0.237	3.640
Diabetes	0.037	2.968	1.067	8.256
Neurological disease	0.773	1.512	0.091	25.173
Stroke	1.000	0.000	0.000	
Hypertension	0.769	1.131	0.496	2.583
Liver disease	0.999	0.000	0.000	
Obesity	0.023	5.363	1.254	22.936
COPD	0.785	1.242	0.261	5.909
Kidney disease	0.360	3.151	0.270	36.797
**Laboratory values**				
Glycaemia (mg/dL)	0.000	1.030	1.017	1.043
Creatine (mg/dL)	0.122	1.633	0.877	3.041

Total bilirubin (mg/dL)	0.250	1.449	0.770	2.725
Leukocytes (x10^9/L)	0.000	1.000	1.000	1.000
Lymphocytes (x10^9/L)	0.484	1.000	1.000	1.000
Neutrophil (x10^9/L)	0.000	1.000	1.000	1.001
Procalcitonin (ng/ml)	0.048	2.527	1.010	6.327
Platelet (x10^9/L)	0.932	1.000	1.000	1.000
CRP (mg/L)	0.091	1.004	0.999	1.008
Ferritin (µg/L)	0.002	1.001	1.000	1.001
D-dimer (mg/L)	0.050	1.000	1.000	1.000
LDH (mmol/L)	0.000	1.007	1.003	1.011

OR—Odss Ratio; CI—Confidence interval.

Table S4. Backward logistic regression.

	Effect	*P* value	OR	CI 95%	
				Lower	Upper
**Intubation or death**	0 blood-group	0.019	0.073	0.008	0.654
	Glycaemia > 134.5 mg/dL	0.003	15.09	2.577	88.32
	Procalcitonin > 0.07 ng/mL	0.017	12.92	1.58	105.99
	Ferritin > 934 μg/L	0.039	6.91	1.1	43.5
	D-dimer > 1814.5 mg/L	0.041	13	1.11	152.04
	HGF > 187.5 pg/ml	0.025	7.38	1.28	42.4

CI—confidence interval; OR—Odds ratio.

Table S5. Bootstrap for Variables in the Equation.

	**B**	**Bias**	**Std. Error**	***p***	**95% CI**	
					**Lower**	**Upper**
0 blood-group	-2.624	-10.322	22.637	0.031	-86.377	-0.265
Glycaemia>134.5 mg/dL	2.714	7.976	19.991	0.003	0.724	80.430
Procalcitonin>0.07 ng/mL	2.559	7.854	18.129	0.001	1.124	65.326
Ferritin>934 μg/L	1.933	3.788	12.909	0.013	0.496	52.612
D-dimer>1814.5 mg/L	2.566	10.785	21.444	0.008	0.109	79.985
HGF>187.5 pg/ml	1.998	6.852	16.286	0.003	0.536	61.567

CI—confidence Interval; B—beta coefficient.
